# Mechanical Properties, Surface Assessment, and Structural Analysis of Functionalized CFRPs after Accelerated Weathering

**DOI:** 10.3390/polym13234092

**Published:** 2021-11-24

**Authors:** Dionisis Semitekolos, Georgios Konstantopoulos, Aikaterini-Flora Trompeta, Craig Jones, Amit Rana, Christopher Graham, Mauro Giorcelli, Alberto Tagliaferro, Elias P. Koumoulos, Costas A. Charitidis

**Affiliations:** 1 Research Lab of Advanced, Composite, Nano-Materials and Nanotechnology (R-NanoLab), School of Chemical Engineering, National Technical University of Athens, 9 Heroon Polytechniou, GR-15780 Athens, Greece; diosemi@chemeng.ntua.gr (D.S.); gkonstanto@chemeng.ntua.gr (G.K.); ktrompeta@chemeng.ntua.gr (A.-F.T.); epk@innovation-res.eu (E.P.K.); 2 The Welding Institute, Granta Park Great Abington, Cambridge CB21 6AL, UK; craig.jones@twi.co.uk (C.J.); Amit.Rana@twi.co.uk (A.R.); chris.graham@twi.co.uk (C.G.); 3 Politecnico di Torino, C.so Duca degli Abruzzi 24, 10129 Torino, Italy; mauro.giorcelli@polito.it (M.G.); alberto.tagliaferro@polito.it (A.T.); 4 Innovation in Research & Engineering Solutions (IRES), Rue Koningin Astridlaan 59B, 1780 Wemmel, Belgium

**Keywords:** CFRPs, accelerated weathering, surface assessment, microcomputed tomography, nanoindentation, mechanical properties

## Abstract

The present study focuses on the effect of two novel carbon fibre surface treatments, electropolymerisation of methacrylic acid and air pressure plasma, on the mechanical properties and structural integrity of carbon-fibre-reinforced composites under operational conditions. Extensive mechanical testing was applied, both in nano- and macro-scale, to assess the performance of the composites and the interphase properties after ultraviolet/humidity weathering. The results of the mechanical assessment are supported by structure, surface, and chemistry examination in order to reveal the failure mechanism of the composites. Composites with the electropolymerisation treatment exhibited an increase of 11.8% in interlaminar shear strength, while APP treatment improved the property of 23.9%, rendering both surface treatments effective in increasing the fibre-matrix adhesion. Finally, it was proven that the developed composites can withstand operational conditions in the long term, rendering them suitable for a wide variety of structural and engineering applications.

## 1. Introduction

In the automotive, aerospace, and construction industry, the majority of the carbon-fibre-reinforced polymers (CFRPs) that are being used are epoxy based matrices, while thermoplastics are getting more attention nowadays [1,2,3]. To increase the performance of epoxy-based CFRPs [4,5,6,7] under operational conditions, various carbon fibre (CF) treatments are currently under investigation [8,9]. In service, the composites are exposed to a variety of conditions, such as moisture, ultraviolet (UV) radiation, and temperature variations, all of which are capable of affecting the properties of composite materials (matrix erosion and microcracking, fibre loss and debonding, void formation) [10,11]. Absorbed moisture has many detrimental effects on material performance since it can cause swelling and degradation of polymer composites. UV, on the other hand, can increase levels of embrittlement; transverse tensile strength has been decreased by 29% during long-term outdoor exposure [12]. In addition, higher temperatures accelerate diffusion rates of moisture and enhance ageing process [13,14]. Weak interfacial adhesion between the CF and the polymer matrix is reported to occur in such composites’ manufacturing, which results in inadequate transfer of consequent stresses [15]. Despite the fact that CFs are not 100% inert—even the unsized CFs have some functional groups (~7–10%) as a result of electrochemical bath treatment—most of CFRPs tend to fail at the interface of the fibre and matrix, while accumulated shear stresses can also cause delamination between the layers [16,17].

A wide variety of surface treatments have been developed by researchers to overcome the adhesion issue [18,19]. Commercially available CFs used for epoxy matrices are often treated with an epoxy sizing to improve adhesion. Electrografting of monomers provides a homogeneous coating of polymer onto the CF surface [20]. This interlayer between fibre and matrix absorbs energy from the cracks, delimiting the stress accumulation and enhancing thus the interfacial adhesion. The electropolymerisation of methacrylic acid (MAA) onto CF fabrics is considered as an effective treatment that can increase the mechanical properties and the adhesion of the fibre-matrix interface, as proven in our previous work [21]. Plasma surface treatment can also be applied on CFs [20,22] or directly on the composites surface [23,24]. The aim of this treatment is to increase the oxygen-containing functional groups on the fibre surface. In particular, air pressure plasma (APP) is a treatment performed at atmospheric pressure, which renders it more useful in large-scale applications since no vacuum is needed; plasma is created in proximity to the fibres and promotes direct functionalisation [22,25].

According to Kumar et al. [12], few studies have focused on the controlled determination of synergistic mechanisms, including UV radiation and condensation, which are predominantly responsible for the degradation during outdoor service. The aim of this work is to study CFRPs environmental deterioration, focusing on the surface properties of the advanced composites as well as the interfacial adhesion of the reinforcement and the matrix. Thus, the effect of two novel surface treatments of CF fabrics (electropolymerisation of MAA and APP treatment) are investigated in this case.

## 2. Materials and Methods

### 2.1. Surface Treatments of CF Fabrics

As reinforcing material, CF fabric G0926 5H Satin was used (HEXCEL Industries, Amesbury, MA, USA), with TENAX E HTA40 E13 6K CF on both warp and weft (weight distribution 50%, nominal weight 375 g/m^2^). The CF fabrics were functionalized with the following techniques.

#### 2.1.1. Air Pressure Plasma

**Figure 1:**
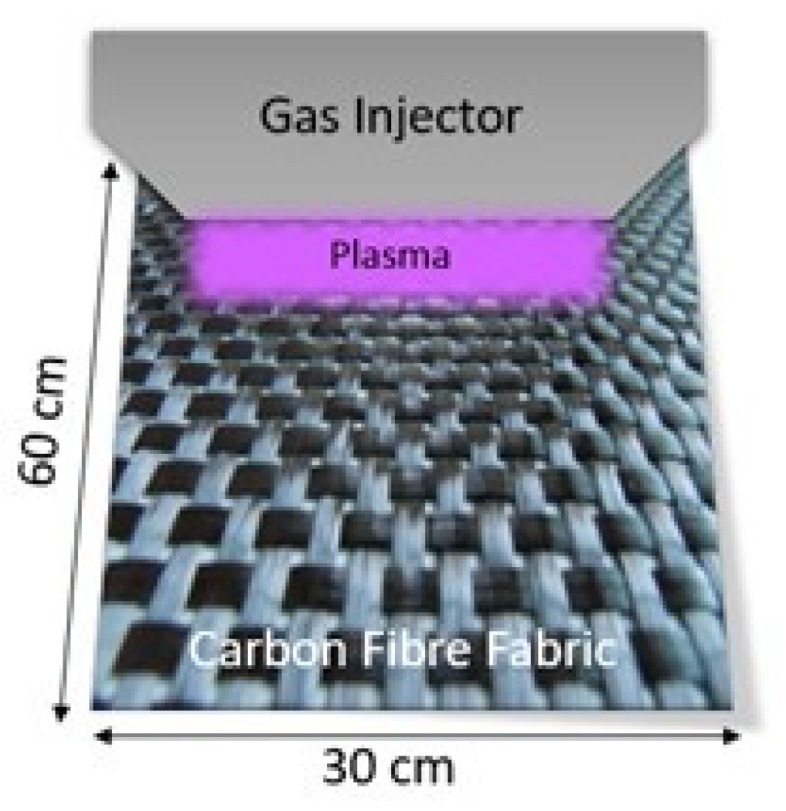
Air pressure plasma treatment representation.

In APP, the woven CF (30 cm × 60 cm) is positioned on an insulative table under a carriage (30 cm × 15 cm) that supports the electrodes that produce plasma. A representation of the APP procedure is depicted in Figure 1. The apparatus consists of a large, flat table used as plate, while the second plate is mobile and able to move horizontally above the first one. The carriage moves on a track to cover all the surface of the fabric at air pressure.

The applied electric field transmits energy to the electrons of the gas, which is then transmitted to the neutral species present on the target by collisions. Elastic collisions slightly raise the kinetic energy of neutral species, while inelastic collisions modify the structure [26]. Plasma treatment is performed on both sides of the woven CF structure (parameters presented in Table 1).

#### 2.1.2. Electropolymerisation of MAA

The electropolymerisation of MAA onto the CF surface is a novel sizing procedure that was initially developed in our previous work [21]. In this study, an optimised procedure was accomplished, able to treat CF fabrics of 30 cm × 30 cm. Prior to electropolymerisation process, all CF fabrics underwent electrochemical modification by cyclic voltammetry. The conditions of electrochemical treatment were selected based on the existing experience of PAN-based CF treatment and removing any pre-existing sizing [27] so as to attach oxygenated groups and increase the roughness. The detailed procedure is presented in the Supplementary Information file. Process parameters are presented in Table 2.

### 2.2. Composite Manufacturing

A three-part resin system (HUNTSMAN industries, Maple Shade, NJ, USA), including epoxy resin (Araldite L Y 556), anhydride hardener (Aradur 917), and an imidazole accelerator (DY 070), was used for composites manufacturing through a vacuum infusion kit (Easy Composites, Stoke-on-Trent, UK). The manufacturing process that was used is vacuum-assisted resin transfer moulding (VA-RTM). Three different types of CFRPs were manufactured; their details are presented in Table 3.

Fibre volume fraction was calculated according to ASTM D3171 [28], which includes two test methods. According to Test Method II, which is applicable only to laminate materials of known fibre areal weight, the reinforcement content by volume, based on the measured thickness of the laminate, can be calculated according to (Equation (1)):

(1) $$V_{r}\left(\%\right)=\frac{A_{r}\;*\;N_{p}*\;0.1}{\rho_{r}*\;h}$$

where A*_r_*: nominal weight of CF fabric (g/m^2^), *N_p_*: number of plies, *ρ_r_*: CF density (g/cm^3^), and *h*: thickness of specimen (mm).

Test Method II omits the void volume, which does not affect the calculations, considering that composite materials contain a negligible porosity, as reported in Section 3.2.

### 2.3. Weathering under Operational Conditions

Τhe composites (3 specimens from each type) were subjected to cyclic UV/condensation in a Q-Lab weathering chamber (QUV) according to the procedure detailed in [29], which reproduces damage caused by sunlight, rain, and dew. UV radiation is generated by eight fluorescent lamps (UV-A region, 340 nm). The intensity of UV radiation is monitored and controlled by four Solar-Eye irradiance detectors, which are calibrated every 500 h. Water condensation is provided by vapour from a water bath, which condenses on the exposed surface of the specimen, simulating dew. This particular test is a cyclic test consisting of 8 h of UV-A exposure at 60 °C, followed by 4 h of condensation at 50 °C. [29]. Every 250 h, testing was interrupted, and samples were removed from the environmental chamber for physical characterisation and surface inspections. Samples were tested over a full period of 1000 h.

### 2.4. Performance Assessment

The surface morphology of the CFRPs was studied using a Nikon Optiphot Differential Interference Contrast (DIC) microscope under 5× and 40× magnification. A FEI Quanta 650 FEGSEM (SEM) up to 4000× magnification was used for the surface examination. Wight Light Interferometry (WLI) was been used at 50× magnification to monitor the extent of matrix degradation after 1000 h QUV-A exposure. The internal structure of the composites [30] was observed by a compact desk-top Bruker micro-CT, 3D X-ray scan system, SkyScan 1272. Specific information for the examination procedure is included in the Supplementary Information. Fourier-transform infrared spectroscopy (FTIR) measurements were conducted to study the surface chemistry of the composites, using an Agilent 4100 handheld spectrophotomer (spectral resolution: 4 cm^−1^, range: 4000–650 cm^−1^). Shear and tensile tests were performed on a tensile machine from MTS Systems Corporation (Eden Prairie, MN, USA) with a maximum load of 100 kN, according to ASTM D2344 [31] and ASTM D638 [32], both before and after weathering for all samples. Five samples of each composite type were tested. Hysitron (Minneapolis, MN, USA) TriboLab^®^ Nanomechanical Test Instrument was used for indentation testing, equipped with a standard Berkovich diamond-shaped tip. Displacement control was set at 200 nm, as described in [33], in accordance to ISO 14577-1:2015.

## 3. Results

### 3.1. Deteriotation Mechanisms of Exposed CFRPs

**Figure 2:**
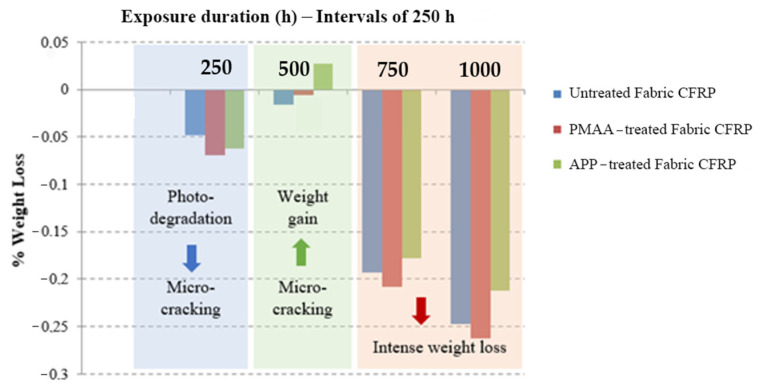
Weight loss data collected for 1000 h QUV.

As a first step to understand the effect of the accelerated weathering to the composite structures, the weight of each specimen was monitored during the entire duration of environmental exposure tests at time intervals of 250 h. The specimens were removed from the QUV chamber specifically during the UV illumination cycle and subsequently weighed on an analytical balance. Figure 2 plots the variation of specimen weight as a function of time.

The overall reduction observed in specimens’ weight was 0.25% for the reference sample, 0.26% for the PMAA-treated fabric CFRPs, and 0.21% for the APP-treated fabric CFRPs after 1000 h of cyclic exposure to UV radiation and condensation. As it can be seen, the highest reduction was for the PMAA-treated fabric CFRP, which indicates that the polymer coating on the CFs can increase the water absorption and thus the overall hydrolysis of the specimen. On the other hand, it was notable that during the micro-cracking phase, the APP-treated CFRPs increased their weight. The plasma treatment introduces oxygen-containing groups on the CFs surface; thus, it is easier to absorb humidity when micro-cracks appear, and CFs are exposed to the atmosphere. The different phases identified during the whole exposure of the specimens in the weathering chamber and the phenomena that take place according to each weight variation are explained in Table 4. From the weight loss reduction rates that were calculated for each case, it is notable that the APP-treated CFRP had the lowest reduction rate at the final phase, despite the fact that it showed the highest weight increase rate at phase 2. It is worth noting that the specimens’ weight was still seen to be decreasing steadily at the end of the test. This means that, at the end, the APP-treated CFRP would show the lowest weight reduction and thus the least deterioration.

In Table 5, the details of the surface morphology of the composites, prior to and after weathering, are presented by utilizing different techniques. Surface spots can be observed in all samples as a result of the laminating process. DIC reveals an imprinted “crisscross” pattern, which is responsible for the surface disruption that is causing reduced surface gloss levels, as shown in the Supplementary Information. The epoxy rich layer was sufficiently eroded after cyclic exposure to UV radiation and condensation, exposing the upper most layer of underlying CFs. Evidence of moisture absorption and micro-cracking can also be seen in the exposed matrix. SEM highlighted how much of the epoxy has eroded, exposing the CFs underneath. WLI revealed that prior to exposure, the apparent surface texture of the bulk material is homogenous but particularly rough. After 1000 h exposure, erosion of the epoxy matrix surface occurred, exposing the fibres from the uppermost layer of CF fabric. Although microscopy alone cannot assess the fibre treatment, it can reveal the level of degradation in the surrounding matrix. This is evidenced by the presence of cracks and exposure of fibres from the fabric layer. This type of degradation of the matrix occurs rapidly (within the first 250 h) as a result of the poor UV resistance of the epoxy resin.

### 3.2. Structural Analysis

Since the composite architecture and properties are directly dependent on the selected manufacturing process of the CFRPs or, in this case, VA-RTM, possible defects, such as pores, misaligned fibres, or resin-rich regions, need to be detected through mCT analysis in order to ensure that all pre-exposed composites are of the same quality. Specifically, the infiltration of the resin during the resin infusion process and the compaction of placed CF fabrics can be evaluated, especially in this study, where the CFs have been treated. The porosity ranges in relatively low values that can be attributed to the vacuum infusion technique. A trend was observed for all types of CFRPs after exposure in the accelerated weathering chamber. More specifically, the total porosity was doubled (Table 6). These results are also confirmed by the surface assessment, which showed increase in roughness of the samples, as mentioned in Section 3.1. Degree of anisotropy was also calculated by CTan using the mean intercept length approach. Anisotropy is defined as a material’s directional dependence of a physical property; hence, it is a characteristic that dwindles as defects are induced on the material.

**Figure 3:**
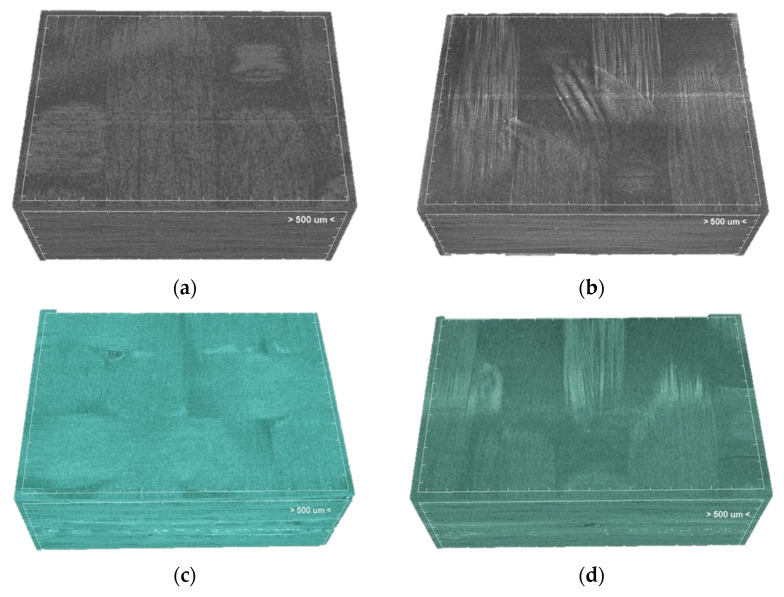

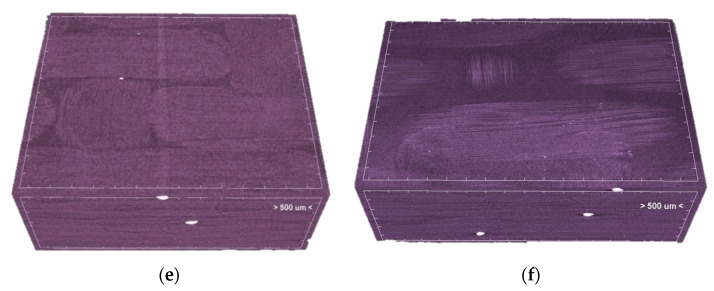
Internal structure of CFRP pre-exposure (**a**) untreated, (**c**) PMAA, and (**e**) APP; post-exposure (**b**) untreated, (**d**) PMAA, and (**f**) APP.

Figure 3 illustrates an indicative volume of the CFRPs of this study before and after their exposure (1000 h), which occurred after the reconstruction of the images during the m-CT analysis. These images confirm all the previous observations about the surface degradation. As mentioned in Supplementary Information, it might be hard to distinguish the epoxy resin with the carbon fibre due to the similar attenuation coefficient during m-CT analysis. However, the resin degradation in the outer layer allows the carbon fibres to be separable and appear vividly. In addition, images of the CFRPs after the exposure in environmental conditions appear to be glossier in comparison to those pre-exposed, indicating the matrix erosion.

### 3.3. Surface Chemistry Examination

**Figure 4:**
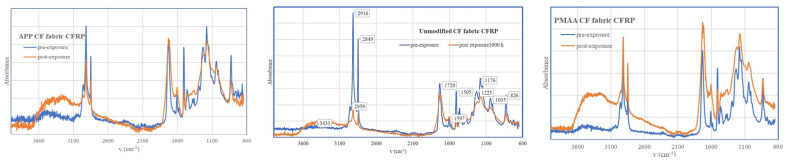
Comparative FT-IR spectrum pre-exposure (blue trace) and post exposure (orange trace).

FT-IR under Attenuated Total Reflectance (ATR) mode was used to study the surface chemistry of the composites prior to and after weathering. The spectra obtained are presented in Figure 4 and the identified peaks in Table 7.

The overall shape of the spectra remains before and after exposure; thus, it can be noted that although degradation occurred, as shown from the surface morphology characterisation, the structure of the composite and the weave of the fabric ensures that there is still sufficient epoxy resin on the surface. Peaks at 2919 and 2850 cm^−1^ are related to C-H stretching of the monomer units, and the broad peak between 3100–3300 cm^−1^ is related to hydroxyl groups. The apparent enlargement of the broad region is a result of increased hydrolysis, as in the case of APP- and PMAA-treated CFRPs. There is a noticeable reduction in the peak intensity at approximately 1500 cm^−1^ for the case of the surface-treated fabrics. This reduction (N-H deformation of amine cross linker) is an indication of absorbance of atmospheric oxygen, which leads to increased embrittlement and micro-cracking. On the other hand, the peak at 1730 cm^−1^ increased in the case of PMAA sample after weathering [34,35].

### 3.4. Mechanical Performance Assessment

#### 3.4.1. Shear Testing

**Figure 5:**
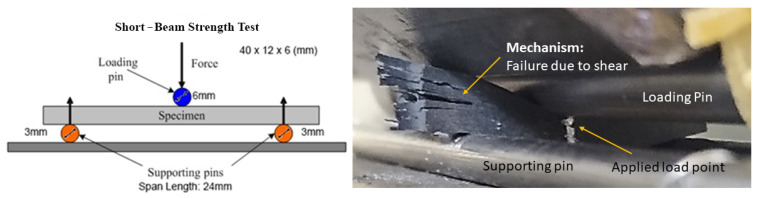
Fracture mechanism observation during short-beam test.

**Figure 6:**
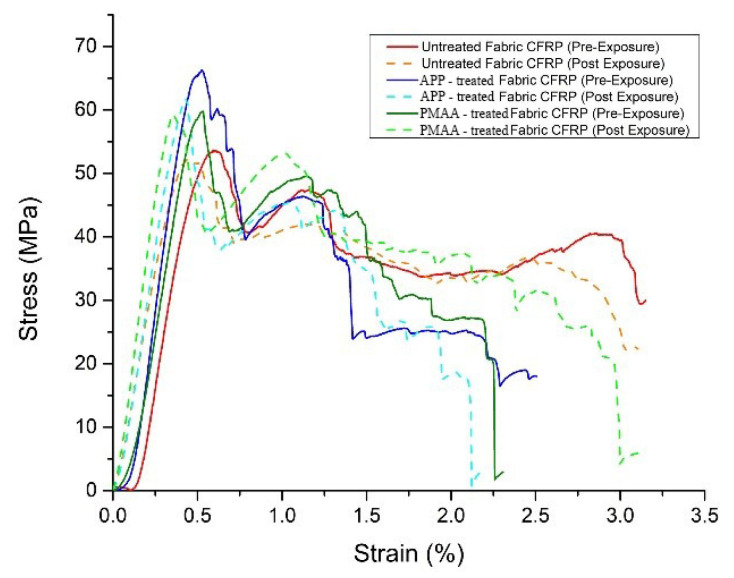
ILSS stress-strain curves for CFRPs prior to and after exposure.

Five specimens were subjected to short-beam tests in order to determine their interlaminar shear strength. Fracture mechanism had to be investigated in order to make sure that specimen’s failure was due to interlaminar shear and not flexural or inelastic deformation. Figure 5 indicates an acceptable failure mechanism in accordance to ASTM D2344; thus, interlaminar shear strength can be calculated and is presented in Table 8 along with the stress-strain curves in Figure 6. CFRPs with PMAA treatment on CF fabrics exhibited an increase of 11.8%, while APP treatment improved the interlaminar shear strength (ILSS) of CFRPs by 23.9%, rendering both surface treatments effective in increasing the fibre-matrix adhesion. Composites after environmental exposure seem to maintain their interlaminar strength; however, conclusions should be derived after tensile test results.

#### 3.4.2. Tensile Testing

**Figure 7:**
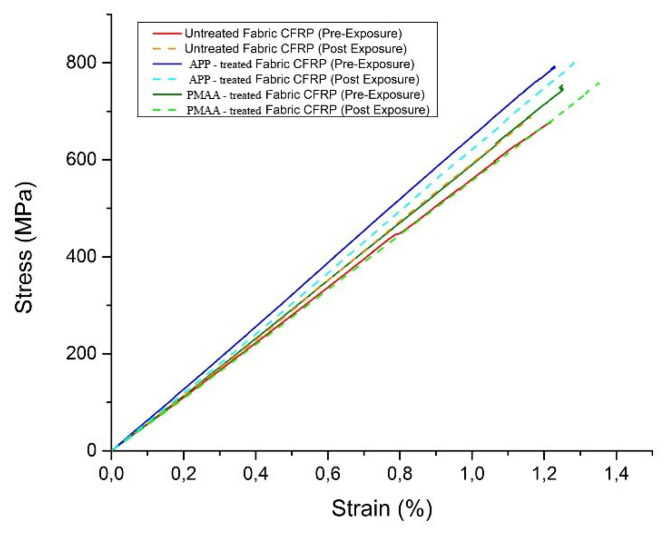
Tensile stress-strain curves for CFRPs prior and after the exposure.

Figure 7 illustrates the stress-strain curves for the CFRPs before and after exposure to environmental conditions. Both surface treatments appear to enhance the tensile strength of the composite materials. The reference CFRP exhibited a tensile strength of 676 MPa, whereas the PMAA-treated fabric CFRP revealed a tensile strength of 754 MPa, an increase of approximately 12%. The most promising results in terms of tensile strength appeared on the APP-treated fabric CFRP, achieving a tensile strength of 797 MPa, corresponding to an increase of approximately 18%. These improvements are attributed to the increase of interlaminar shear strength (ILSS), as reported above. An effective surface treatment on the CF can optimize the hydrogen bonding between the epoxy active groups and the modified CF, which in turn results in a CFRP with increased interfacial shear strength (IFSS) [21]. This is also supported by the SEM fracture analysis reported in Section 3.4.3. Low interfacial bond strength promotes large-scale debonding around broken fibres and reduces their load-carrying capacity. On the other hand, high interfacial bond strength tends to extend cracks transversely into the matrix at fibre breaks. An optimum IFFS induces the highest tensile strength of composites. The same behavior regarding the mechanical performance is preserved to the composites after the exposure with the highest tensile strength on the APP-treated fabric CFRP 803 MPa (+17% compared to the reference CFRP) and 751 MPa for the PMAA-treated fabric CFRP (+10% compared to the reference CFRP).

The most notable highlight is that the CFRPs maintain their ultimate strength after 1000 h of QUV. Table 9 summarizes the mechanical properties (tensile strength, young modulus, elongation) for the three types of composite materials under investigation.

Comparing the pre-exposure to the post-exposure results, it is shown that the degradation is limited in the outer matrix layer of the CFRP without penetrating in the CF layers, thus leaving the mechanical properties of the CFRPs unaffected. This also confirms that an effective wet treatment like electropolymerisation and a plasma treatment at atmospheric pressure can only have a positive effect in the mechanical properties of composite materials without any deterioration on performance during their service life. In the study of Kumar et al. [12], it was also observed that after 1000 h of cyclic exposure to both UV radiation and condensation, commercial CFRP specimens manufactured for aerospace and rotorcraft structures presented no longitudinal tensile strength deterioration. However, it can be expected that over long periods of environmental exposure, the synergism between UV and condensation will cause damage to the load transfer between fibres and matrix due to the erosion of the latter, leading to a catastrophic structural failure of the composite.

#### 3.4.3. Study of Fractured Surface

**Figure 8:**
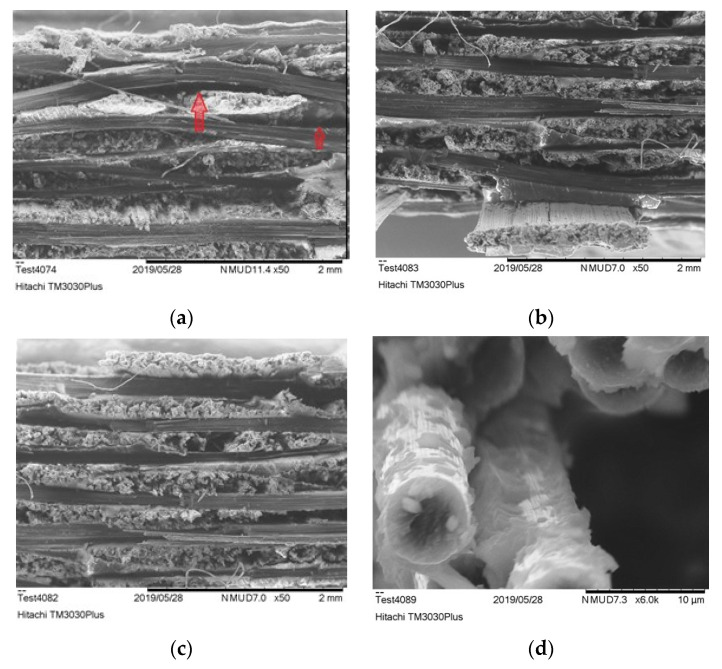
SEM fracture analysis: (**a**) Untreated fabric CFRP (**b**) PMAA-treated fabric CFRP (**c**) APP-treated fabric CFRP (**d**) Epoxy resin bonded on CF after APP treatment.

In order to investigate the connection of the increased tensile strength with the improved IFSS, the CFRPs cross-section after the tensile testing was observed through SEΜ [36] for all samples pre-exposed to the weathering; all images in Figure 8 depict the fractured surface, which is perpendicular to the axial load. Figure 8a depicts the cross-section of the reference CFRP. It is visible that the composite has suffered serious delamination during its stress since large holes are evident between the CF tows (red arrows). In Figure 8b,c, on the other hand, the cross-section of PMAA—(b) and APP (c)-treated fabric CFRP seems unaffected by delamination, maintaining the parallel fibre orientation. Since the APP treatment led to increased tensile strength, it was worthy to examine the interphase of the functionalised CF in detail. Hence, in Figure 8d, a large quantity of epoxy resin on the exposed APP-treated surface of the fractured fibre is observed. These findings indicate the optimized hydrogen bond between epoxy resin and CF, resulting in increased IFSS.

#### 3.4.4. Mapping of Nanohardness

**Figure 9:**
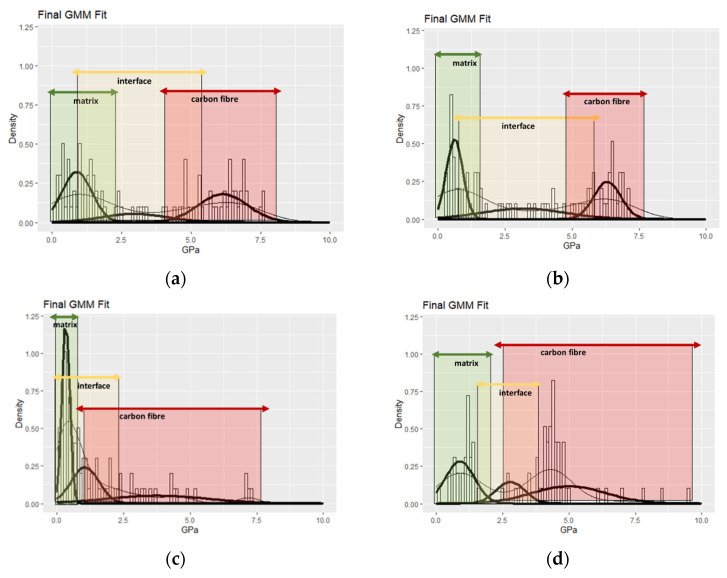

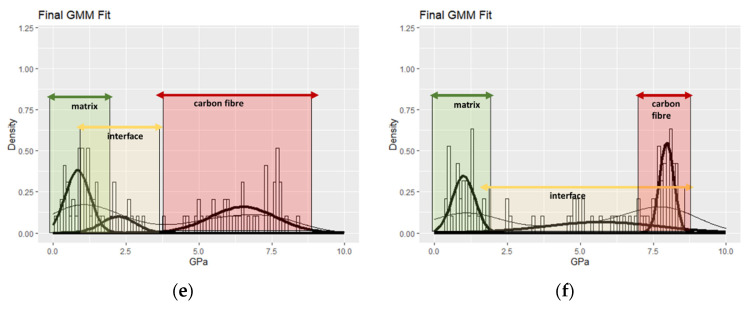
Phase quantification with Probability Distribution Fitting (PDF) of nanoindentation hardness for (**a**) pristine, (**b**) W-pristine, (**c**) PMAA, (**d**) W-PMAA, (**e**) APP, (**f**) W-APP specimen.

In order to evaluate the nanohardness of the modified CFRPs, nanoindentation analysis was performed separately, accompanied with probability distribution analysis (PDA) that facilitates the phase deconvolution of the examined area. A normal Gaussian distribution was applied for fitting the histograms of hardness for each specimen tested. Number of phases is identified by monitoring the histogram density, and the optimum solution is obtained by expectation maximisation (EM) algorithm [37]. In this way, the results of nanomechanical mapping are quantitatively assessed, and possible bias of the local packing density is taken into consideration. The fitness function is presented in Equation (2):

(2) $$\mathrm{PDF}=\frac{1}{\sqrt{2\pi }\cdot \sigma }\exp\left(-\frac{{\left(H-\mu \right)}^{2}}{2\sigma^{2}}\right)$$

where H accounts for hardness (GPa) and is the independent variable in Equation (2), and μ and σ correspond to the mean value and standard deviation, respectively. The fitting is illustrated in Figure 9.

Nanohardness values derived by PDF fitting (Figure 9) are in accordance to relevant reported values in literature [38,39,40,41]. The utilisation of an expectation maximisation algorithm enabled to obtain a global solution regarding the mean values of each phase identified out of a sample of 100 observations for each specimen in order to obtain a statistical representation of the nanomechanical behavior.

The weathering temperature conditions fall below the glass transition of the epoxy resin [29], and consequently, improvement in hardness due to curation reactions was demonstrated in matrix phase for all weathered specimens [42]. The quantified interfacial properties of pristine and APP-treated CFRPs demonstrated a wider range of values in comparison to PMAA-modified CFRPs; in case of PMAA composites, a small variation was observed, and also a reduction of hardness was evidenced, especially for the epoxy matrix and the interface, due to the nature of PMAA modification, as indicated by the observation of the condition prior to and after weathering. More specifically, the adopted modification conditions are expected to create a wider interphase [43], with a reduced content in crystalline PMAA sequences and may act as vulnerabilities upon exposure to weathering conditions [44]. Plasma-treated CFRPs demonstrated a higher mean hardness value for the interphase compared to the other specimens, which could be connected to the reduction of the available interphases for initiation of degradative reactions [45].

It is worth to note that important changes were evidenced in CFs phase, which are influenced by the stereochemistry of the interphase bonding [44], the matrix contribution to the nanomechanical response, and also the exact coordinates of the contact of the indenter and the carbon fibre surface. In case of the pristine specimen exposure of CFRPs in the UV and heat/humidity conditions did not degrade the standard interface of unmodified CFs; the increment in the mean value of hardness falls within the standard deviation of the pristine specimen. Plasma functionalisation provided the most promising results, and carbon fibre chemical adhesion with the matrix proved to be even more resistive to indentation loading compared to the as-manufactured state [43]. Actual improvement in hardness by 6.1% due to APP modification was evidenced compared to the reference specimen and, after 1000 h of total exposure to corrosive/erosive conditions (both hydrothermal, and UV ageing), exceeded by 26.1% the hardness of the weathered pristine specimen. It led to enhanced overall results by outperforming all specimens tested, and this can be attributed to low penetration depth of plasma on CFs surface; thus, CFs did not suffer any degradation effect [45]. Moreover, the interphase variation is increased after weathering, and also obtained the highest reported value amongst the tested specimens. This is a special feature that facilitates the harmonic stress transfer upon weathering, and as a result, it provides hydrothermal durability [44,46].

## 4. Conclusions

An experimental investigation was conducted in this study in order to characterize the physical, chemical, and mechanical degradation of the CFRPs that have been manufactured via VA-RTM by using CF fabrics with two surface functionalisations, electropolymerisation of MAA and APP, which were performed with the aim of improving the CF adhesion with the epoxy matrix. Interestingly, both surface treatments provided composites that exhibited an increased tensile strength, 12% and 18%, compared to untreated fabric. In order to study the effect of UV radiation and water condensation on the composite materials, the specimens were subjected to cyclic UV/condensation in a Q-Lab weathering chamber. Physical degradation mechanisms, such as micro-cracking, were identified by monitoring weight loss or gain, showing that the APP-treated specimens would withstand better in the long term. Micrographic observations of the composite surface, through SEM, DIC, and WLI, proved that physical degradation occurs on the CFRP surface, where the CFs are revealed after the resins’ erosion. In addition, the degradation chemistry of the epoxy resin was examined using FTIR spectroscopy. All CFRPs studied maintained more of less same mechanical properties after cyclic weathering of 1000 h. In fact, APP treated CFRPs lost approximately 7% of ILSS vs. 2% for untreated CFRPs and 0.7% for PMMA-treated CFRPs despite a significant increase in ILLS in pre-exposure conditions. The tensile properties did not change at all based upon the data presented and the standard deviations. The results of this assessment proved that CFRPs manufactured with surface treated CF fabrics maintained their ultimate strength after 1000 h of cyclic UV/condensation, leading to the conclusion that the degradation of the composite material appears to be localized at the external surface, while the structure of the composite remains unaffected, which was also confirmed by images and 3D analysis performed by m-CT. Finally, the interphase properties of the functionalised CFs and the epoxy matrix were investigated through nanohardness measurements after weathering. The severity of dynamic contact-induced damages and their interactions at both surface and interphase regions led to the conclusion that APP modification provides not only enhanced weathering durability but also demonstrated actual and up to 26.1% improvement in carbon fibre phase hardness derived by dynamic nanoindentation. This is an indication for improved resistance to plastic deformation and also increased resistance to matrix debonding in case of failure. The next steps should be focused on prolonged exposure or harsher conditions to reveal possible deterioration of mechanical properties.

## Figures and Tables

**Table 1:** Parameters for APP functionalisation.

Plasma Power	Carriage Speed	Total Number of Passages (for Each Side)	Distance Electrodes—Sample	Carrier Gas (In Proximity of Plasma)
500 W	5.4 m/s	30	2.05 mm	Argon

**Table 2:** Parameters and conditions for CFs electrochemical treatment and electropolymerisation.

Electrochemical Treatment				Electropolymerisation				
Aqueous Solution	Potential (V)	Number of Cycles	Scan Rate (V/s)	Monomer Concentration (M)	Potential (V)	Electrolyte Concentration (M)	Crosslinker Concentration (mM)	Electro-polymerisation time (s)
5% H_2_SO_4_	−3 to +3	10	0.1	0.3	−0.435	0.4	10	3600

**Table 3:** Conditions and specifications of manufactured CFRPs.

Specimen Type	Matrix	Matrix Weight Mixing Ratio	Reinforcement	Fibre Volume Fraction (%)
Untreated Fabric CFRP	Araldite LY 556 + Aradur 917 + Accelerator DY 070	100:90:0.5	G0926	56.3%
APP-treated Fabric CFRP			APP-treated G0926	56.8%
PMAA-treated Fabric CFRP			PMAA-treated G0926	56.6%

**Table 4:** Degradation phases and weight loss reduction rate.

Phase	Range (h)	Phenomenon	Observation	Weight Loss Reduction Rate (h^−1^)		
				Ref	PMAA	APP
1	0–250	Photo degradation that leads to micro-cracking	Initial decrease in weight	−0.00019	−0.00028	−0.00025
2	250–500	Micro-cracking that permits increase in moisture ingress [10]	Weight gain	+0.00013	+0.00025	+0.00036
3	>500	Removal of material from the surface of the specimens [12] (Confirmed also by DIC and WLI, Section 3.1, Table 5).	Intense weight loss compared to phase 1	−0.00071	−0.00081	−0.00082
				−0.00022	−0.00022	−0.00014

**Table 5:** Surface morphology assessment after 1000 h of accelerated weathering.

Sample	DIC		SEM	WLI	
	Pre-Exposure	Post Exposure	Post Exposure	Pre-Exposure	Post Exposure
Unmodified fabric CFRP	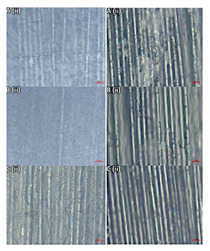		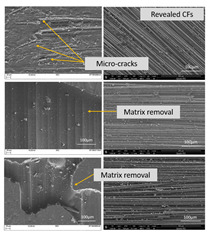	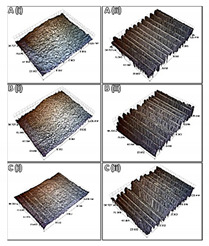	
APP fabric CFRP					
PMAA CFRP					

**Table 6:** Results of 3D analysis performed by m-CT.

	Pre-Exposure				Post Exposure			
Specimen Type	Degree of Anisotropy	Open Porosity	Closed Porosity	Total Porosity	Degree of Anisotropy	Open Porosity	Closed Porosity	Total Porosity
Untreated Fabric CFRP	3.28	0.0019	0.0234	0.0253	4.31	0.0203	0.0256	0.0459
APP-treated Fabric CFRP	3.91	0.001	0.0122	0.0132	4.33	0.0115	0.0129	0.0244
PMAA-treated Fabric CFRP	3.38	0.0021	0.0185	0.0206	3.56	0.0156	0.0195	0.0351

**Table 7:** Identification of peaks from FT-IR spectra [32,33].

Band (cm^−1^)	Assignment
3100–3600	O–H stretching
~3000	Stretching of C–H of the oxirane ring
2919, 2850	Stretching C–H of CH_2_ and CH
1730	Ester group
1368	Deformation CH_3_ of C–(CH_3_)_2_
1176	Stretching C–O–C of ethers
1035	Stretching C–O of oxirane group
826	Stretching C–O–C of oxirane group
759	Rocking CH_2_

**Table 8:** ILSS of composite materials pre- and post-exposure (Number of specimens tested: 5).

	Pre-Exposure	Post Exposure
Specimen Type	ILSS (MPa)	ILSS (MPa)
Untreated Fabric CFRP	53.5 ± 3.1	52.4 ± 3.2
APP-treated Fabric CFRP	66.3 ± 3.5	61.7 ± 3.3
PMAA-treated Fabric CFRP	59.8 ± 3.4	59.4 ± 3.4

**Table 9:** Tensile properties of composite materials pre- and post-exposure. (Number of specimens tested: 5).

	Pre-Exposure			Post Exposure		
Specimen Type	Tensile Strength (MPa)	Young Modulus (GPa)	Strain (%)	Tensile Strength (MPa)	Young Modulus (GPa)	Strain (%)
Untreated Fabric CFRP	676 ± 17.5	55.0 ± 4.3	1.21	685 ± 19.5	55.1 ± 3.8	1.2
APP-treated Fabric CFRP	797 ± 16.9	61.4 ± 3.8	1.25	803 ± 17.6	61.9 ± 4.1	1.25
PMAA-treated Fabric CFRP	754 ± 15.7	56.9 ± 3.3	1.28	755 ± 16.3	55.9 ± 3.2	1.32

## Data Availability

The raw/processed data required to reproduce these findings cannot be shared at this time due to technical or time limitations.

## Supplementary Materials

The following are available online at https://www.mdpi.com/article/10.3390/polym13234092/s1, Figure S1: Surface gloss measurements for 1000 h QUV-A, Table S1: m-CT Scanning Conditions
