# Supplementary material for: Mechanical Properties, Surface Assessment, and Structural Analysis of Functionalized CFRPs after Accelerated Weathering
Source: Polymers (Basel). 2021 Nov 24;13(23):4092. doi: 10.3390/polym13234092 (PMC8658828; doi:10.3390/polym13234092)
Supplement: Supplementary file 1 [file polymers-13-04092-s001.zip › polymers-1444207-supplementary.pdf]

polymers

MDPI

Supplementary Materials

Mechanical Properties, Surface Assessment and Structural Analysis of Functionalized CFRPs after Accelerated Weathering

Dionisis Semitekolos [1], Georgios Konstantopoulos [1], Aikaterini-Flora Trompeta [1], Craig Jones [2], Amit Rana [2], Christopher Graham [2], Mauro Giorcelli [3], Alberto Tagliaferro [3], Elias P. Koumoulos [1,4], Costas A. Charitidis [1,*]

[1] Research Lab of Advanced, Composite, Nano-Materials and Nanotechnology (R-NanoLab), School of Chemical Engineering, National Technical University of Athens, 9 Heroon Polytechniou, GR-15780, Greece, diosemi@chemeng.ntua.gr (D.S); gkonstanto@chemeng.ntua.gr (G.K.); ktrompeta@chemeng.ntua.gr (A.F.T)

[2] The Welding Institute, Granta Park Great Abington, Cambridge CB21 6AL UK, craig.jones@twi.co.uk (C.J); Amit.Rana@twi.co.uk (A.R); chris.graham@twi.co.uk (C.G)

[3] Politecnico di Torino, c.so Duca degli Abruzzi 24, 10129 Torino, Italy, mauro.giorcelli@polito.it (M.G); alberto.tagliaferro@polito.it (A.T)

[4] Innovation in Research & Engineering Solutions (IRES), Rue Koningin Astridlaan 59B, 1780, Wemmel, Belgium epk@innovation-res.eu (E.P.K)

* Correspondence: charitidis@chemeng.ntua.gr; Tel.: +30-2107724030

Citation: Semitekolos, D.; Konstantopoulos, G.; Trompeta, A.-F.; Jones, C.; Rana, A.; Graham, C.; Giorcelli, M.; Tagliaferro, A.; Koumoulos, E.P.; Charitidis, C.A. Mechanical Properties, Surface Assessment, and Structural Analysis of Functionalized CFRPs after Accelerated Weathering. *Polymers* **2021**, *13*, 4092. https://doi.org/10.3390/polym13234092

Academic Editors: Stana Kovačević and Ivana Schwarz

Received: 15 October 2021
Accepted: 19 November 2021
Published: 24 November 2021

Publisher's Note: MDPI stays neutral with regard to jurisdictional claims in published maps and institutional affiliations.

Copyright: © 2021 by the authors. Licensee MDPI, Basel, Switzerland. This article is an open access article distributed under the terms and conditions of the Creative Commons Attribution (CC BY) license (https://creativecommons.org/licenses/by/4.0/).

Electropolymerization of MAA

The electropolymerization of MAA onto the CF surface is a novel sizing procedure that has been developed in our previous work. In this study, a scaled-up procedure has been accomplished and CF fabrics of 30cm x 30cm can be now functionalised. Prior to the electropolymerization process, all CF fabrics undergone electrochemical modification by cyclic voltammetry, in order to attach oxygenated groups and increase fabrics roughness. For this pre-treatment, CF fabrics, which are considered as the working electrode, are placed in a 3L electrochemical cell filled with aqueous solution of 5% wt. H_2SO_4. The working electrode is interposed between two metallic stainless-steel plates that act as auxiliary electrodes while the system is completed by the reference electrode (saturated silver chloride electrode (Ag/AgCl, KCl(sat))). To achieve the electrochemical oxidation, ten potentiometer scans are performed (Voltage range: -3V to 3V, scanning rate: 0.1V/s).

After washing with deionised water, CF fabrics are re-placed in the electrochemical cell to continue with the electropolymerization procedure. The electrochemical cell in this case is filled with aqueous solution of 0.3M MAA (monomer), 0.4M ZnCl2 (electrolyte) and 10mM N,N'-methyl-bis-acrylamide (cross-linker). The electropolymerisation lasts for 3600 s and runs at a potential of -0.435V (potential required to reduce the monomer).

Examination procedure via microcomputed tomography

Micro computed tomography (m-CT) can provide a list of quantitatively characteristics and images of the internal structure of composites. The internal structure was observed by a compact desk-top Bruker micro-CT, 3D X-ray scan system, SkyScan 1272. The system consists of a microfocus sealed X-ray source which operates at 20-100kV and 10W (<5μm spot size @ 4W), an X-ray detector with a maximum resolution of 11Mp (4032x2688 pixels) and a 14bit cooled CCD fibre optically coupled to scintillator. Table S1 summarizes the scanning conditions of all CFRPs samples.

Table S1. m-CT Scanning Conditions.

**Voltage**	40kV
**Current**	166μA
**Filter**	Al 0,25mm
**Pixel Size**	9μm
**Resolution**	1344 x 896

The measurement of specimens in the Sky Scan 1272 CT requires the appropriate sample geometry. For this reason, the composite specimens were cut with a wheel at the required dimensions: 1cm x 0.7cm x 0.3cm (LxWxT). Samples were placed centered and aligned with the rotation axis on top of the holder. The holder was subsequently mounted on a rotational stand in the scanner chamber. A rotation step of 0.3° with 180o tomographic rotation was selected to obtain the 2D x-ray images. 2D images of cross-sectional slices were reconstructed via N Recon Reconstruction software (Bruker).

The composite materials studied in this article consist of epoxy resin and CF, two components that are largely carbon-based and have a similar attenuation coefficient. As a consequence, to be able to distinguish those two phases, the voltage range was set in a relative low value.

Gloss Measurements

The surface gloss of the samples was measured with Elcometer 407 Statistical Glossmeter. For the purpose of this study, measurements at 60° were performed. As the surface of the CFRP begins to degrade and the matrix erodes, any changes in the surface morphology or exposure of CFs will cause the surface roughness and ultimately the observed gloss to alter. In Figure S1 it can be observed that the onset of surface degradation, witnessed by the changes in gloss happens quite quickly (after 200 h). The gloss level increase can be attributed to the initial surface micro-roughness of the exterior matrix layer, since initial gloss levels of the non-weathered matrix were measured to be generally low (10-15 GU, compared to 90 GU for a high gloss surface). This data coincides with WLI imagery which shows the exterior surface to be pitted and rough. This micro-structure has been imparted from the mold during the vacuum forming process, since the surface of the composite was not polished as post-treatment. As the weathering continues, the weave of the composite material from the uppermost layer of fabric becomes exposed alongside with areas of polished epoxy, effectively bringing out this apparent gloss increase. If the testing were to continue beyond 1000 h, then the remaining surface epoxy would degrade sufficiently to expose further layers of CF fabric causing the gloss reading to fall.

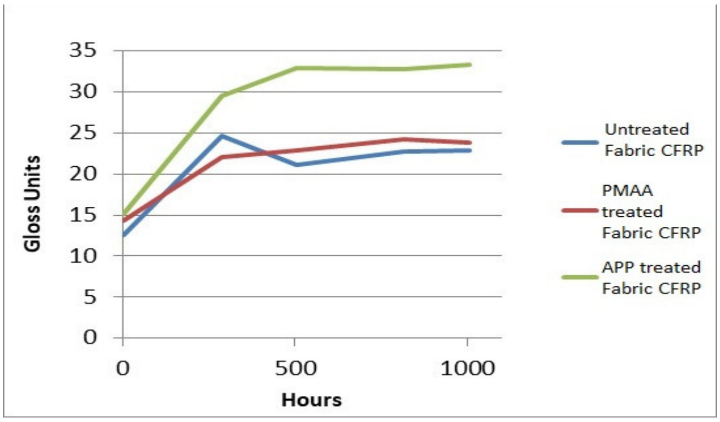

Figure S1: Surface gloss measurements for 1000 h QUV-A.
